# Spatial Variations in Vitreous Oxygen Consumption

**DOI:** 10.1371/journal.pone.0149961

**Published:** 2016-03-01

**Authors:** Karthik Murali, Dongyang Kang, Hossein Nazari, Nicholas Scianmarello, Enrique Cadenas, Yu-Chong Tai, Amir Kashani, Mark Humayun

**Affiliations:** 1 Department of Biomedical engineering, University of Southern California, Los Angeles, California, United States of America; 2 Department of Ophthalmology, University of Southern California, Los Angeles, California, United States of America; 3 Department of Pharmacology and Pharmaceutical Sciences, University of Southern California, Los Angeles, California, United States of America; 4 Department of Electrical Engineering, California Institute of Technology, Pasadena, California, United States of America; 5 University of Southern California, Eye Institute, Los Angeles, California, United States of America; 6 University of Southern California, Institute of Biomedical Therapeutics, Los Angeles, California, United States of America; Massachusetts Eye & Ear Infirmary, Harvard Medical School, UNITED STATES

## Abstract

We investigated the spatial variation of vitreous oxygen consumption in enucleated porcine eyes. A custom made oxygen source was fabricated that could be localized to either the mid or posterior vitreous cavity and steady state vitreous oxygen tension was measured as a function of distance from the source using a commercially available probe. The reaction rate constant of ascorbate oxidation was estimated *ex vivo* by measuring the change in oxygen tension over time using vitreous harvested from porcine eyes. Vitreous ascorbate from mid and posterior vitreous was measured spectrophotometrically. When the oxygen source was placed in either the mid-vitreous (N = 6) or the posterior vitreous (N = 6), we measured a statistically significant decrease in vitreous oxygen tension as a function of distance from the oxygen source when compared to control experiments without an oxygen source; (p<0.005 for mid-vitreous and p<0.018 for posterior vitreous at all distances). The mid-vitreous oxygen tension change was significantly different from the posterior vitreous oxygen tension change at 2 and 3mm distances from the respective oxygen source (p<0.001). We also found a statistically significant lower concentration of ascorbate in the mid-vitreous as compared to posterior vitreous (p = 0.02). We determined the reaction rate constant, k = 1.61 M^-1^s^-1^ ± 0.708 M^-1^s^-1^ (SE), of the oxidation of ascorbate which was modeled following a second order rate equation. Our data demonstrates that vitreous oxygen consumption is higher in the posterior vitreous compared to the mid-vitreous. We also show spatial variations in vitreous ascorbate concentration.

## Introduction

The vitreous, while being the largest structure in the eye, has historically been regarded as possessing limited active functions for vision. It was thought to be required only to ensure a clear optical pathway for light and for maintaining intraocular pressure. However, ophthalmology researchers over the past 25 years have expanded the body of basic and clinical science pertaining to the vitreous in health as well as in disease[1,2]. Today, we know that the vitreous is essential for maintaining molecular and mechanical homeostasis of the eye.

One critical role of the vitreous involves oxygen homeostasis in the eye. Yu, Linsenmeier and others performed measurements of intravitreal and intraretinal oxygen tension in various species in health as well as disease conditions [3–7]. As the field of vitreous surgery developed, Stefansson et al extensively studied intraocular oxygen before and after vitrectomy and showed that oxygen gradients flatten out after vitrectomy[8]. It was initially suggested that the change in oxygen diffusion coefficient, due to the change in vitreous fluid viscosity, explained the differences in intraocular oxygen levels between vitrectomized and non vitrectomized eyes[9]. However, further studies showed that vitreous cavity fluid itself consumes oxygen in an ascorbate dependent manner[10,11]. The decrease in ascorbate concentration after vitrectomy emphasized the active role healthy vitreous plays in maintaining the intraocular oxygen environment. In addition, the non-homogenous nature of the vitreous humor suggests that it might have site specific features[12]. This was supported by a recent investigation into the metabolic signature of the vitreous humor which indicated that ascorbate was unequally distributed across various topological areas within the vitreous[13]. Despite all these elegant studies, the reaction kinetics of the intravitreal ascorbate reaction are not known and we also do not fully understand the spatial and temporal characteristics of vitreous oxygen consumption. Further study of oxygen consumption by the vitreous is required to fully understand these phenomena and develop appropriate therapies to replace oxygen supply.

Intravitreal oxygen therapy has been proposed as a treatment for retinal ischemia[14]. For these types of treatments, a better understanding of the spatial and temporal characteristics of vitreous oxygen consumption is essential. In this paper, we take the first step towards that goal by investigating the spatial dynamics of vitreous oxygen consumption in porcine cadaver eyes. The reaction kinetics of the ascorbate-oxygen reactions are studied via measuring the decay of oxygen tension in vitreous samples. The spatial characteristics of vitreous oxygen consumption are studied through intravitreal oxygen measured from an oxygen source.

## Methods

For the following experiments, we used fresh porcine cadaver eyes (Sierra Medical Science) that were shipped and used for experiments at USC with 6 hours of harvesting. These specimens included only the eyeball with minimal amounts of periocular adventitial tissue and typically did not include the muscle attachments or any other components of the orbit. Only whole globes from 6 month old pigs were used for these studies. This ensured that the globes had fully formed vitreous with high gel content and no liquefaction.

### Oxygen Source Fabrication

A custom made device was used in order to create a constant focal source of oxygen within the cadaver eye’s vitreous humor. The oxygen source device fabrication has two main steps: mold fabrication and silicone casting. The mold was fabricated through negative dry film photoresist (DuPont) laminated on a fresh silicon wafer. It was patterned via UV light exposure and developed. For the casting of silicone (MED4-4210, two-part, medical-grade, NuSil Technology LLC) are mixed at a 10:1 ratio by weight, degassed under vacuum, and applied onto the patterned mold. A hollow stainless steel tube is inserted through the neck of the oxygen source device to ensure that air permeates through the base of the oxygen source device and only exits through the tip of the oxygen source device. This semi-permeable oxygen source device acts as a conduit of air from the base to the tip and effectively acts as point source of oxygen within the vitreous[15].

### Intravitreal Oxygen Diffusion Measurements

**Fig 1:**
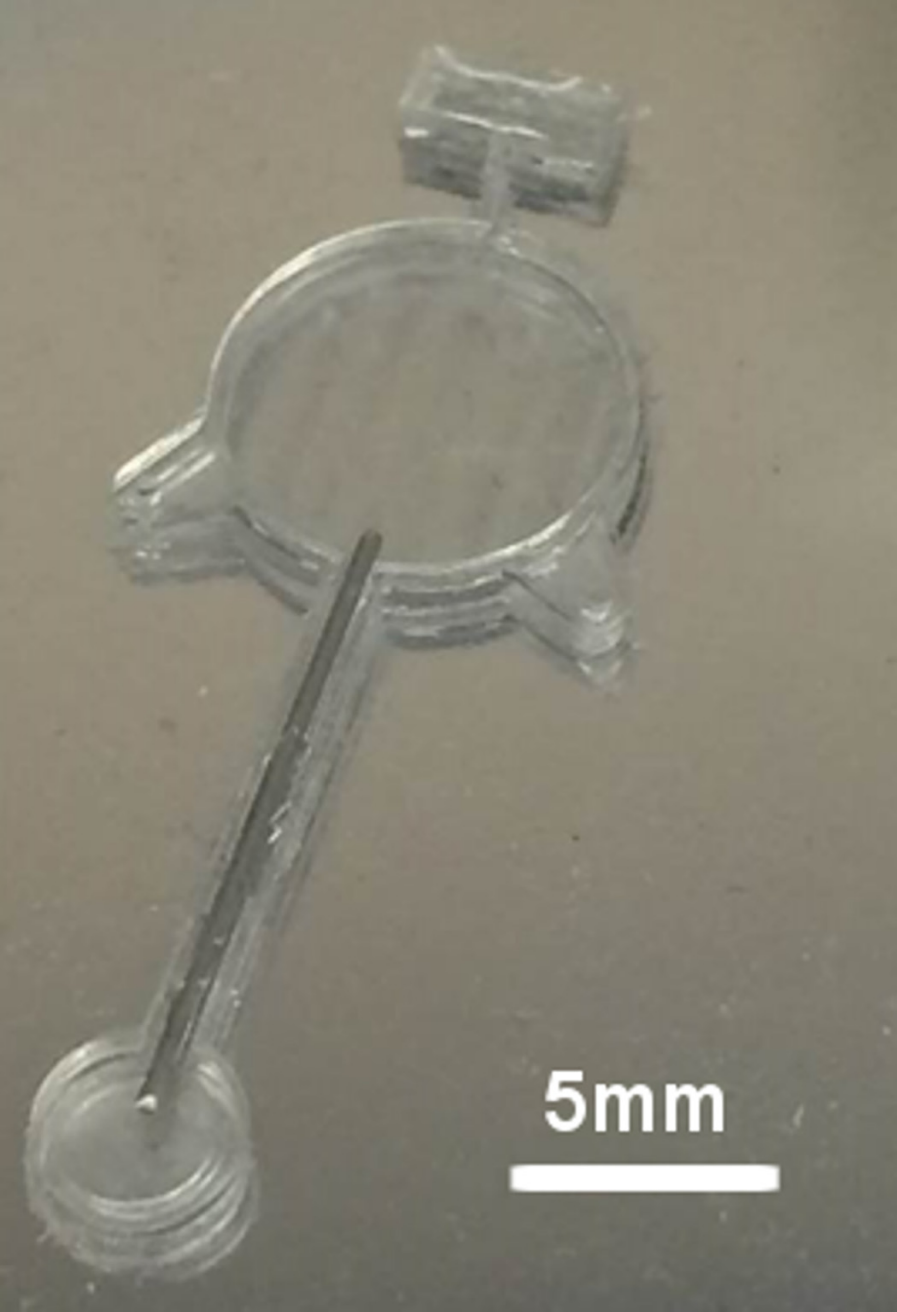
Oxygen source device. The base is 10mm in diameter and the ti is 4mm in diameter. The hollow stainless steel tube connects the base to the tip. Only the base and tip are permeable to oxygen.

**Fig 2:**
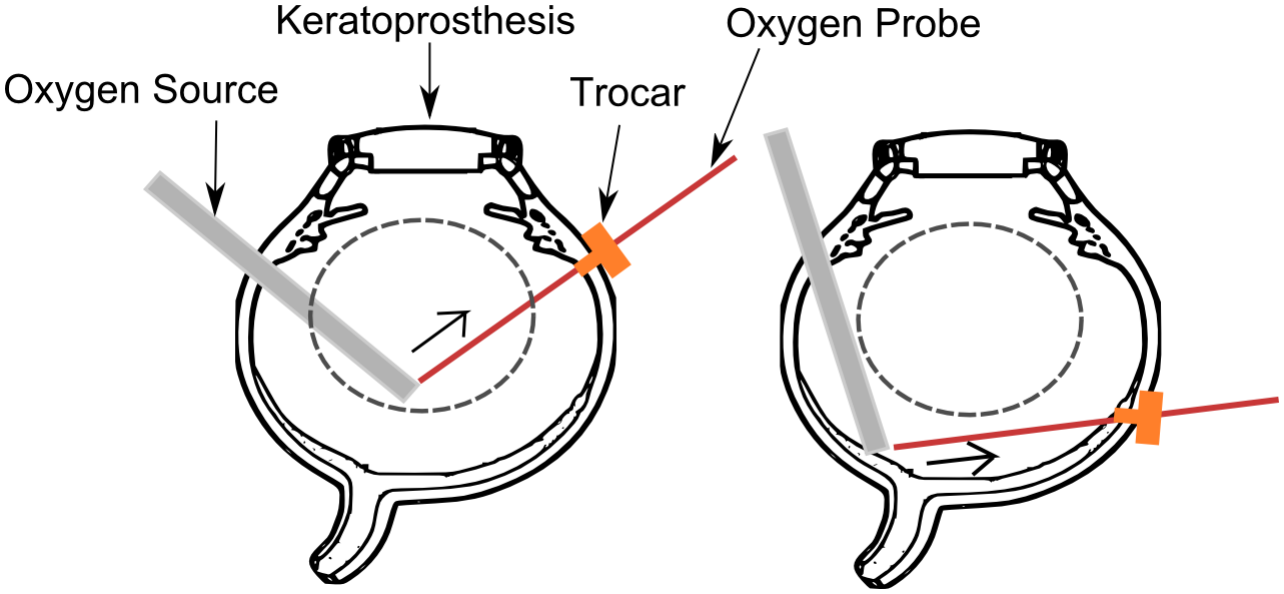
*Ex vivo* porcine eye preparation and intravitreal oxygen measurement methods. Left: Oxygen source is positioned in the mid-vitreous and the oxygen probe is retracted in the direction shown by the arrow. Right: Oxygen source is positioned in contact with the retinal tissue and the oxygen probe is retracted in the direction shown by the arrow. The probe is positioned such that it stays in the posterior vitreous region. The trocar is used to facilitate oxygen probe entry and retraction without creating any motion artefact. The dashed lines indicate regions that are designated has mid vitreous and posterior vitreous.

In order to ensure visibility into the vitreous of the porcine cadaver eyes, the native cornea and the lens were removed and replaced with a keratoprosthesis (Ocular Instruments). Next, the custom made oxygen source shown in Fig 1 was placed so that the tip of the oxygen source device was either located in the mid-vitreous or posterior vitreous. The location of the tip was approximated by the length of the cannula inside the eye. The base of the oxygen source was left outside the globe and exposed to atmospheric air. A schematic is shown in Fig 2. Next, a 23 gauge valved trocar (Alcon) was implanted 180° across the semi-permeable oxygen source. This positioning allowed for placement of a commercially available oxygen measuring probe (Oxford Optronix, OxyLab), here on referred to as oxygen probe, in proximity of the oxygen diffuser. The 2 positions of the trocar are illustrated in Fig 2. We inserted the oxygen measuring probe through the trocar and positioned it at various distances from the oxygen source. For our control experiments, the oxygen source was removed after probe was positioned. At the start of the experiment, the probe was positioned 0mm from the surface of the oxygen source tip. The eyes were then left in a dark environment for an hour for the oxygen diffusion to reach steady state. After an hour, recordings were performed. At each distance location, oxygen tension is recorded for 1.5 minutes. In order to verify that the probe was 0 mm from the surface of the oxygen source tip, we measured oxygen tension at the surface of the oxygen source tip and ensured that it was equal to that of air. We used a micromanipulator (Edmund Optics, Linear translational stage) to retract the probe to desired distances (0, 1, 2, 3, 4mm) away from the oxygen source tip. The oxygen probe has a resolution of 0.1mmHg and an accuracy of ±10% when partial pressure of oxygen (pO_2_) is below 150mmHg and ±20% when the pO_2_ is above 150mmHg.

### Vitreous Oxygen Consumption Rate Measurements

We dissected vitreous humor from several porcine cadaver eyes and placed them in a beaker. Lens material, retinal tissue, and choroid tissue were carefully removed during the dissection procedure. We used a magnetic stirrer to homogenize the vitreous for 5 minutes. This ensured that the oxygen tension in the vitreous was equal to that of air (160mmHg). Vitreous samples were collected with a vitrector (Alcon Constellation Vision System). The vitreous was then transferred to a clear glass beaker and covered with a rubber stopper. The glass vial was tightly sealed with a crimper (IVPACKS LLC). Care was taken to minimize the pocket of air within the beaker. We introduced the oxygen probe through the rubber septum with an 18 gauge needle and recorded the decay of vitreous oxygen tension until oxygen tension reached 10mmHg. This is representative of the physiological levels of oxygen in the vitreous. At the end of the experiment, vitreous was again collected with a vitrector and the ascorbate concentration differences in the samples were analyzed. In a few samples, ascorbate oxidase was added to the vitreous sample. Ascorbate oxidase reduces ascorbate present in the vitreous, slowing down vitreous oxygen consumption.

### Ascorbate Measurements

**Fig 3:**
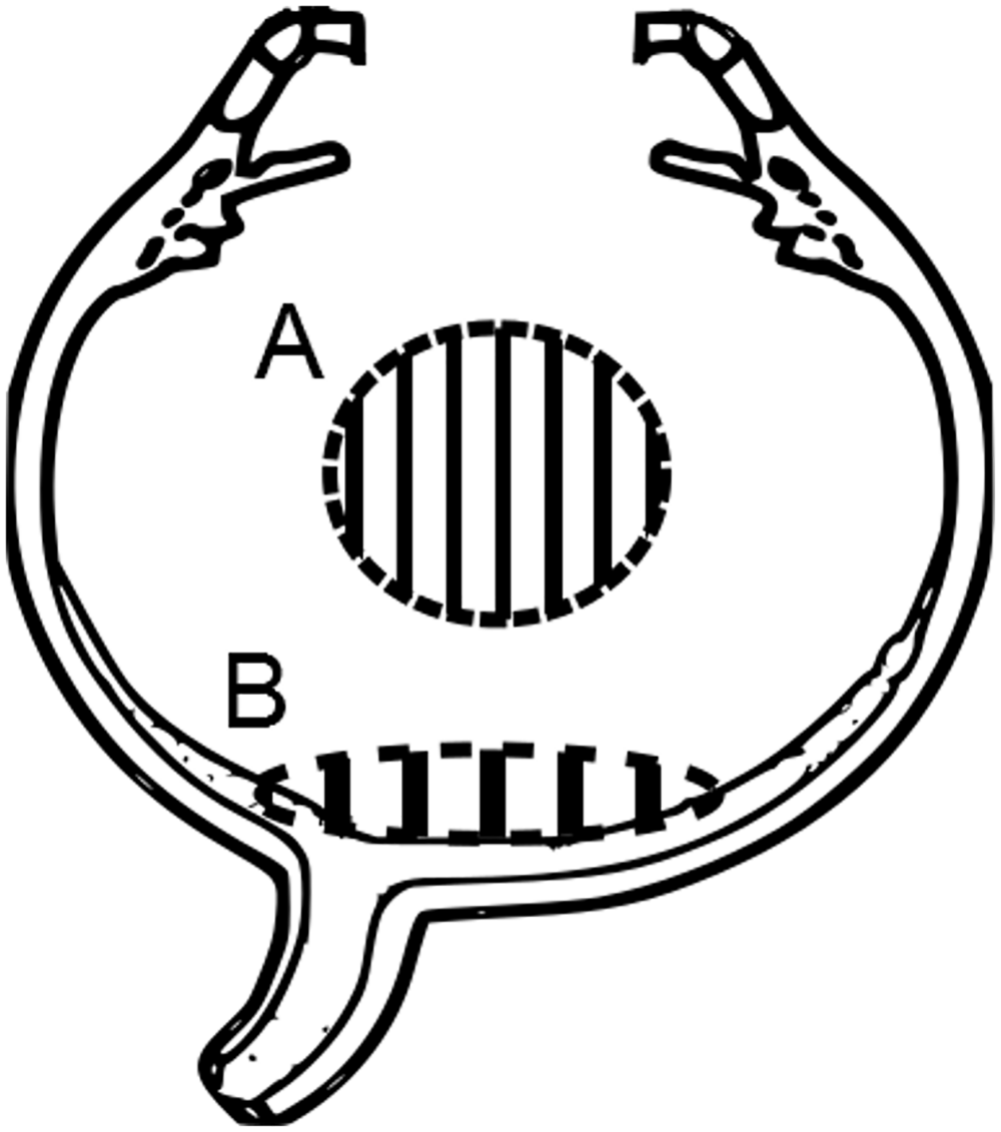
Illustration of vitreous regions that were biopsied for ascorbate measurements. (A) The site of the mid-vitreous sample collection. (B) The site of the posterior vitreous sample collection.

We carefully dissected out samples from the vitreous core and posterior vitreous of porcine cadaver eyes. To ensure visibility into the vitreous, we removed the cornea, anterior lens capsule and extracted the lens leaving the posterior lens capsule intact. We attached a 3cc syringe to a vitrector (Alcon Constellation Vision System) and inserted it into the eye via a 23 gauge trocar. With a cut rate of 5000 cuts per minute, we first aspirated 200 μl samples from the core (Fig 3). Following that, samples from the posterior vitreous were aspirated. Care was taken to not cut the retinal tissue.

We tested these samples in duplicate with an ascorbic acid assay kit (Sigma Aldrich MAK075). In this assay, ascorbic acid concentration is determined using the Ferric Reducing/Antioxidant and Ascorbic Acid (FRASC) assay. In this assay, Fe^3+^ is reduced to Fe^2+^ by antioxidants present in the sample, which results in a colorimetric (593 nm) product. The addition of ascorbate oxidase to parallel samples oxidizes any ascorbic acid present allowing for the measurement of the ascorbic acid concentration. A freshly prepared standard curve was used for all measurements. The specificity of this method for ascorbate has been previously validated using gas chromatography-mass spectroscopy[10].

## Results

**Fig 4:**
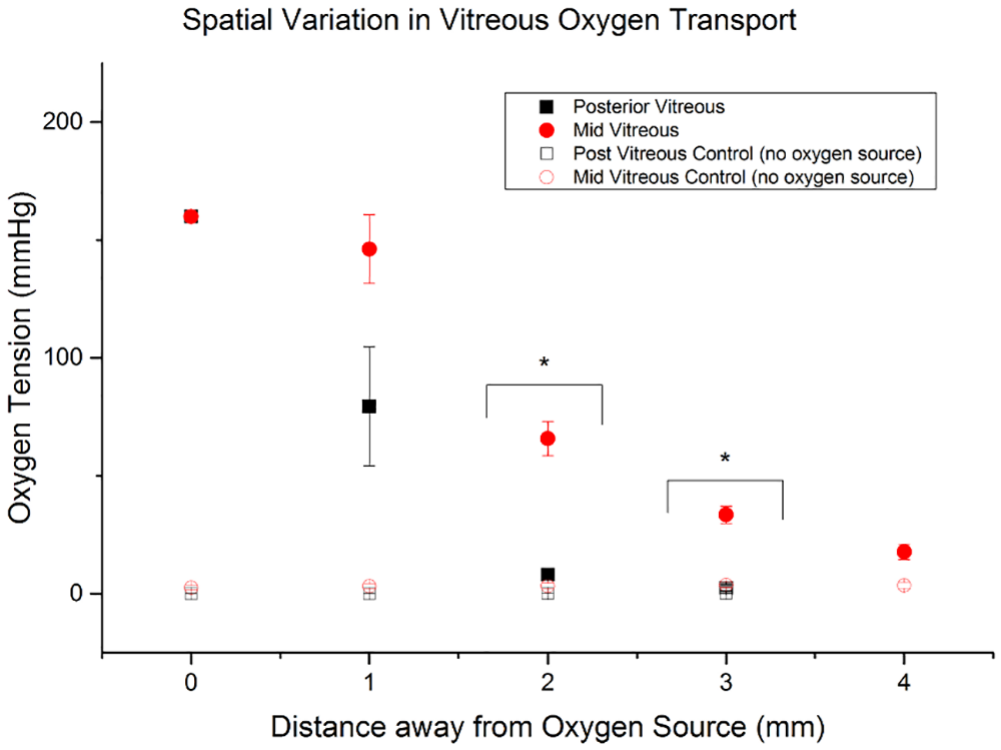
Comparison in oxygen diffusion kinetics between mid and posterior vitreous. Black Squares: Oxygen tension with increasing distance from an oxygen source in the posterior vitreous. Red Circles: Oxygen tension with increasing distance from an oxygen source in the mid-vitreous. Oxygen tension recordings are higher in the mid-vitreous compared to the posterior vitreous as distance from the oxygen source increases. Black Squares: Oxygen tension recordings with increasing distance from the probe in the absence of any oxygen source in the posterior vitreous. Hollow Circles: Oxygen tension recordings with increasing distance from the probe in the absence of any oxygen source in the mid-vitreous. Hollow Squares: Oxygen tension recordings with increasing distance from the probe in the absence of any oxygen source in the posterior vitreous. Please note that the open symbols are not clearly identifiable because they overlap in the graph.

In 6 eyes, the oxygen source was placed in the mid-vitreous (vitreous core) and in another 6 eyes it was placed in the posterior vitreous. Each of the control groups had 4 eyes. When the oxygen probe was 0mm away from the oxygen source, it measured the pO_2_ of air which was 160mmHg. In the presence of an oxygen source, mid-vitreous pO_2_ and posterior vitreous pO_2_ measurements are higher across all distances as compared to the control pO_2_ measurements (Fig 4). A two-sided, two-sample t-test was conducted to statistically compare pO_2_ recordings between experiments with an oxygen source and control experiments. When the oxygen source was placed in the mid-vitreous, there was a statistically significant decrease in the oxygen tension with distance from the oxygen source versus control; (p<0.005 at all distances 0, 1, 2, 3, 4mm). When the oxygen source was placed in the posterior vitreous, there was a statistically significant decrease in the oxygen tension with distance from the oxygen source versus control as well; 0mm (p<0.01), 1mm (p = 0.01), 2mm (p<0.01), and 3mm (p = 0.018) distances.

Overall, oxygen tension recordings at all distances from the oxygen source were higher when the oxygen source was placed in the mid-vitreous as compared to when it was placed in the posterior vitreous (Fig 4). A two sided, two-sample t-test was conducted to compare pO_2_ recordings between mid-vitreous and posterior vitreous experiments with an oxygen source. The mid-vitreous oxygen tension was significantly higher when compared to the posterior vitreous oxygen tension values at distances 2 and 3mm from the oxygen source (p<0.001).

We modeled our experimental system as a point-source of oxygen diffusing radially in spherical coordinates. Using Fick’s laws of diffusion, we obtain Eq (1), where C is the concentration of oxygen in M (Molar), r is distance away from the source in mm, and R is the rate of oxygen consumption by the vitreous in Ms^-1^, and D is the diffusion coefficient of oxygen in water at 20°C (0.00197 mm^2^/s).

$$\frac{dC}{dt}=\frac{D}{r^{2}}\frac{d}{dr}\left(r^{2}\frac{dC}{dr}\right)+R$$ (1)

Under steady state conditions, Eq (1) can be reduced to Eq (2). This assumption is valid because experimentally, the oxygen tension at various distances at the time of measurement (after 1 hour time lapse) was constant. Also based on the characteristic diffusion time constant, the diffusion process would have reached steady state.

$$\frac{D}{r^{2}}\frac{d}{dr}\left(r^{2}\frac{dC}{dr}\right)=-R$$ (2)

**Fig 5:**
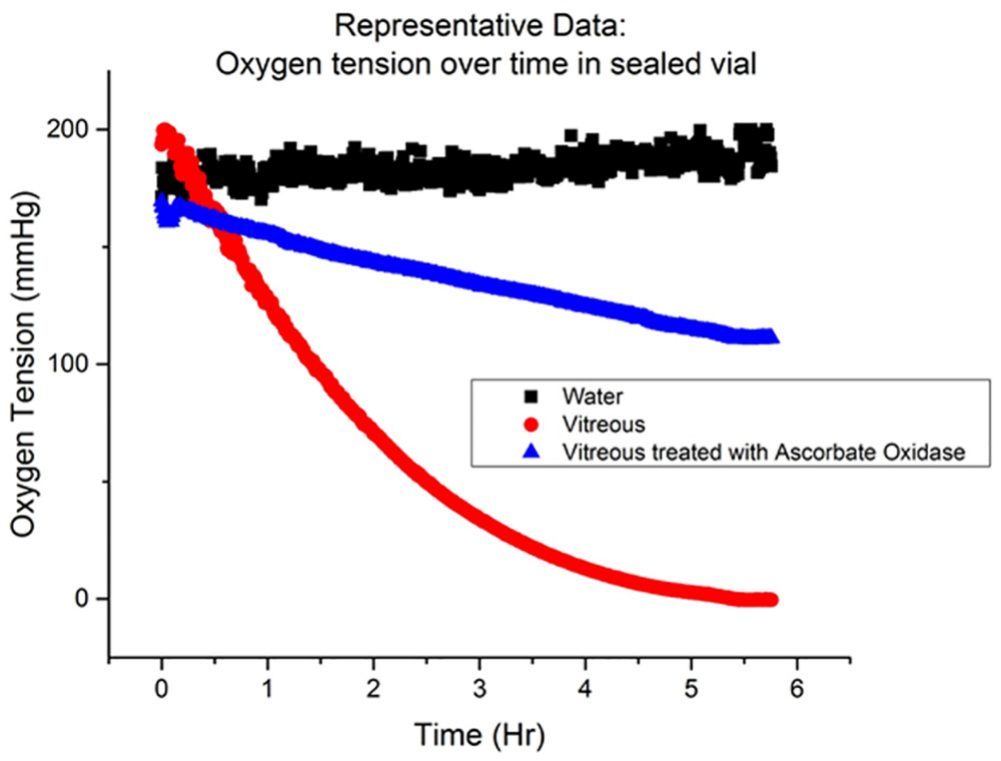
Kinetics of oxygen consumption in vitreous samples (red circle) compared to water (black square) in a sealed chamber and to vitreous treated with ascorbate oxidase (blue triangles).

In order to determine an appropriate reaction rate equation for R, we measured the oxygen consumption rate by the vitreous as described in the methods section and illustrated in Fig 5.

Over the course of ~5.5 hours, the oxygen content in vitreous humor decayed from 160mmHg to 0mmHg. Water was used as a control and the oxygen tension of water remained at baseline levels throughout the duration of the experiment.

The ascorbate concentration in the vitreous humor was measured before and after the experiment described in Fig 5, using the assay described in the methods section. The ascorbate content did not decrease by more than 5% of the initial value. A two-sided, paired, sample t-test was conducted to determine if ascorbate content changed over the time during which vitreous oxygen levels were found to decrease. We could not find a significant difference between the ascorbate content before and after the experiment at α = 5%.

The reaction of ascorbate with oxygen is described in Eq (3). Eq (3) is not meant to suggest a direct two electron transfer from *AH*^*−*^ to *O*_2_ in order to generate *H*_2_*O*_2_. The overall reaction involves 2 one electron transfers (Eqs 3a and 3b). The oxidation of ascorbic acid has been described as following a general solution of the second order rate equation as described in Eq (4)[16].

$$AH^{-}+O_{2}+H^{+}\to A+H_{2}O_{2}$$ (3)

$$AH^{-}+O_{2}^{-.}+H^{+}\to A^{-.}+H_{2}O_{2}$$ (3*a*)

$$A^{-.}+O_{2}\to A+O_{2}^{-.}$$ (3*b*)

$$R=\frac{dO_{2}}{dt}=-k\left[AH^{-}\right]\left[O_{2}\right]$$ (4)

Since the ascorbate concentration in our experimental setup did not change beyond 5%, it was assumed to be constant. Thus, the rate law Eq (4) can be combined with Eq (5) to yield a single effective rate constant as described in Eq (6).

$$\frac{d[AH^{-}]}{dt}=0$$ (5)

$$\text{ln}\left[O_{2}\right]=\text{ln}{\left[O_{2}\right]}_{0}-\overset{´}{k}t$$ (6)

$$where\;\overset{´}{k}=k\left(AH^{-}\right)$$

**Fig 6:**
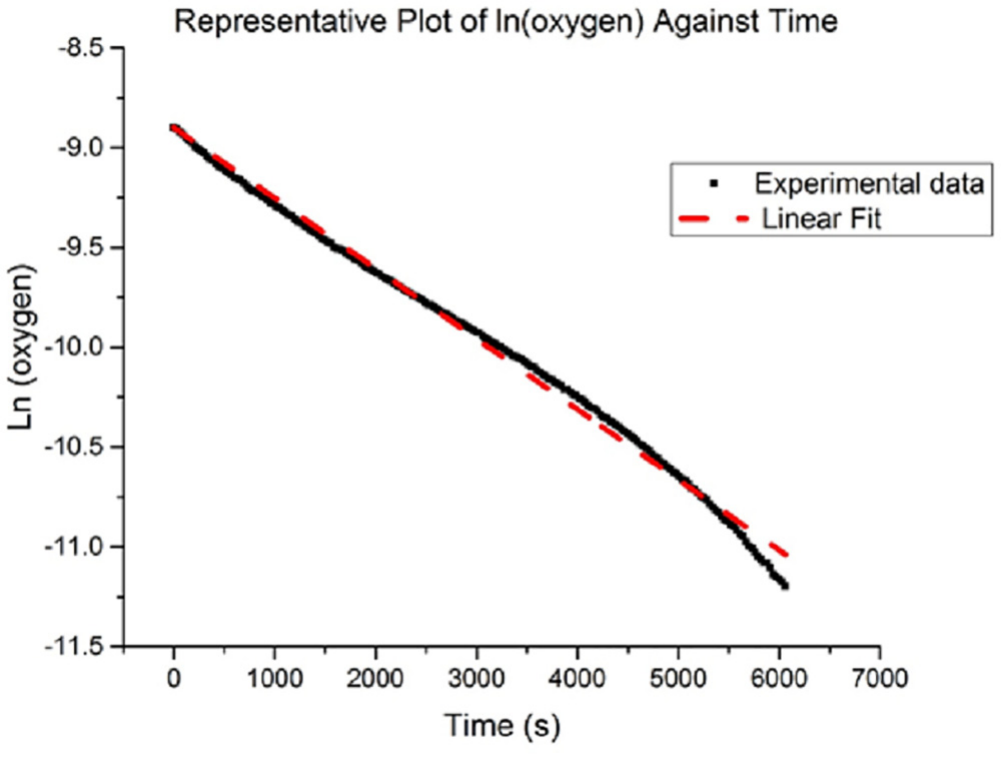
Representative results. Plot of ln (oxygen) against time.

Fig 6 is a plot of ln [O_2_] against time. By fitting ln [O_2_] against time, we can obtain the effective reaction rate constant $\overset{´}{k}$ from the slope of the fitted line. The experimental value for $\overset{´}{k}$ is 1.95*10^-4^s^-1^ ± 4.55*10^-5^s^-1^ (SE). By accounting for the ascorbate concentration in each sample, we obtain the final reaction rate constant k = 1.61 M^-1^s^-1^ ± 0.708 M^-1^s^-1^ (SE).

With the reaction term equation known, we combined Eqs (4) and (2) to obtain a differential that can be solved analytically to produce Eq (7). See supporting information for detailed solution.

$$C\left(r\right)=\frac{c_{1}e^{-r\sqrt{\frac{AH^{-}k}{D}}}}{r}+\frac{c_{2}e^{r\sqrt{\frac{AH^{-}k}{D}}}}{r\sqrt{\frac{AH^{-}k}{D}}}$$ (7)

To explain the spatial variation in vitreous oxygen consumption, we hypothesized that the ascorbate concentrations varied spatially. So, we measured the ascorbate content in the posterior vitreous and compared it with the ascorbate content in the mid-vitreous (Table 1).

**Table 1:** Ascorbate concentration differences between mid-vitreous and posterior vitreous.

Eye	Ascorbate content in mid- vitreous (mM)	Ascorbate content in the posterior vitreous (mM)	Difference in ascorbate content (mM)
1	0.271	0.453	0.182
2	0.321	0.418	0.098
3	0.172	0.384	0.212
4	0.245	0.234	-0.011
5	0.301	0.39	0.088

Vitreous samples of the vitreous core and posterior vitreous from 5 eyes were obtained and analyzed for ascorbate content. A one-sided paired sample t-test was conducted to determine the effect of vitreous location on ascorbate content. There was a significant difference between the ascorbate content in the vitreous core compared to the ascorbate content in the posterior vitreous (p = 0.02). Ascorbate content is higher in the posterior vitreous as compared to the core. The mean ascorbate content in the mid-vitreous and posterior vitreous was 0.262mM and 0.376mM respectively.

**Fig 7:**
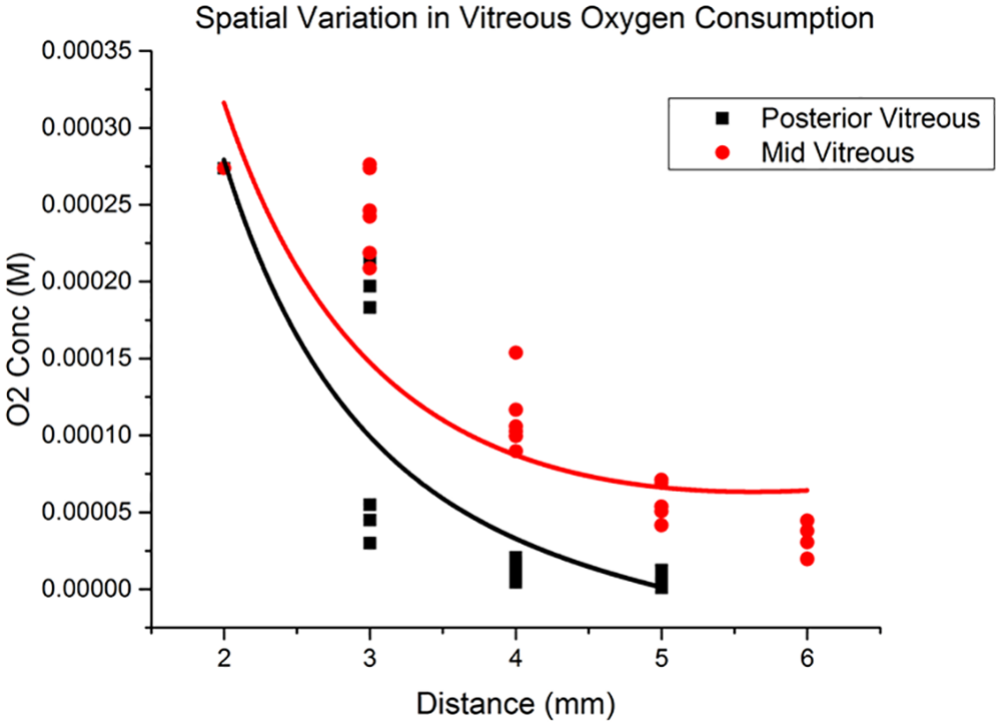
Spatial variation in vitreous oxygen consumption. Experimental data with fitted curve.

These ascorbate and reaction rate constant (k) values were substituted into Eq (7) and the resulting curve was fitted against the experimental data (Fig 7). The oxygen source was modeled as a 2mm radius sphere. Distance from the oxygen source was modeled from the surface of the 2mm radius sphere. Oxygen diffusion coefficient D was assumed to be a constant[17]. Because the vitreous is approximately 99% water, we can assume the oxygen diffusion coefficient to not differ significantly between the mid and posterior vitreous. Coefficients C_1_ and C_2_ were obtained for the mid and posterior vitreous based on the best fit curve (OriginLab). This was done by minimizing the Chi-squared value, which is the square root of the sum of the squares of the distance of each data point from the theoretical curve (Table 2).

**Table 2:** Coefficients and goodness-of-fit indicator for curve fitted to both mid-vitreous and posterior vitreous data.

	C_1_	C_2_	Adjusted R^2^
Mid-Vitreous	0.00148	8.43*10^−6^	0.703
Posterior Vitreous	0.00175	-42	0.86

## Discussion

Oxygen distribution in the eye is tightly regulated. This is evident by the hypoxic environment of ocular tissues, such as the lens (3–9mmHg) and trabecular meshwork (12mmHg) [3,10,18]. Studies have shown that this regulation is performed by vitreous humor [10,18]. In healthy eyes, oxygen from the retinal vasculature in the anterior surface of the retina diffuses into the vitreous humor. There is a gradient in oxygen content from the retina (~22mmHg) to the posterior lens (~9mmHg) which indicates oxygen consumption by the vitreous[11]. Shui and colleagues experimentally demonstrated that vitreous reacts with molecular oxygen via an ascorbate dependent reaction [10]. Ascorbate (Vitamin C) is essential for many processes, such as synthesis of collagen, maintaining prosthetic metal ions in their reduced forms, and scavenging free radicals to prevent oxidative damage to tissues[19]. Ascorbate is present in the vitreous humor in relatively high concentrations and plays an important role in preventing oxidative damage caused by free radical formation during solar radiation [20]. Shui et al’s recent finding suggests that ascorbate’s role in maintaining a low intraocular oxygen environment is equally important. It is interesting to note that humans and other animal species such as guinea pigs and primates are limited to whatever ascorbate they can obtain from their diet since they do not have the ability to synthesize ascorbate from glucose[21].

The ascorbate reaction with molecular oxygen has been studied in a variety of fields [16,22]. Eq (3) is the proposed overall stoichiometric biochemical equation. However, it is important to note that this reaction only occurs as 2 one electron transfer reactions as described in Eqs (3a) and (3b). This reaction will only occur in the presence of catalysts such as light, free radicals, and transition metals; these catalytic reactions are not fully understood [22–24]. Thus the reaction kinetics have not been fully detailed either. Shui et al initially proposed a constant rate of vitreous oxygen consumption. Filas et al later proposed a hyperbolic function (similar to Michaelis-Menton Kinetics) to describe the reaction kinetics[17]. However, based on our empirical evidence, we believe that the reaction kinetics can be best described by a second-order reaction rate law.

While we found a statistical difference in the ascorbate content between the vitreous core and posterior vitreous, ascorbate alone might not be the sole cause of the spatial variation in vitreous oxygen consumption. The concentration of the above mentioned catalysts might also account for the spatial variation in vitreous oxygen consumption. It is important to note that the spatial variation in vitreous oxygen consumption was shown in enucleated porcine eyes, used within 6 hours of enucleation. Despite a short postmortem interval, retinal degeneration might affect the posterior vitreous oxygen consumption. More work needs to be done in vivo to confirm the spatial variation in vitreous oxygen consumption.

It is well established that ascorbate accumulates in the eye of humans and animals at a concentration that is several times higher than that present in the blood plasma. Prior studies have shown that ascorbate contents in the pig’s vitreous, aqueous and blood plasma are 0.28mM, 0.57mM, and 0.01mM respectively [21,25,26]. This is especially important because ascorbate’s high concentration makes it the dominant antioxidant over Superoxide Dismutase[27]. We know that ascorbate does not enter the vitreous by diffusion alone and its intravitreal concentration is maintained by sodium dependent ascorbate transporter (SLC23A2). This transporter is present in the pigmented layer of the ciliary epithelium [28]. Locci et al experimentally demonstrated that ascorbate is found in higher concentrations near the basal area of the vitreous, the part of the vitreous closest to the ciliary body [13]. Locci’s study, together with our findings of higher ascorbate content in the posterior vitreous suggests that ascorbate concentration in the eye exists along a gradient. We hypothesize that ascorbate might enter the eye via the retinal pigment epithelium[29]. We also hypothesize that the posterior vitreous in close proximity to the vitreous cortex, with its denser network of collagen fibers, holds more ascorbate as compared to the vitreous core. Ascorbate’s molecular mass is more than 10x that of oxygen. This spatial variation in ascorbate might account for the spatial variation on vitreous oxygen consumption.

Our findings of a spatial variation in vitreous oxygen consumption might also give us new insight into the results of recent intravitreal oxygen studies. Quiram et al showed that intravitreal oxygen tension in animals with posterior vitreous detachment (PVD) along with vitreous liquefaction is significantly higher when compared to oxygen levels after vitreous liquefaction without PVD. However, oxygen levels in animals with vitreous liquefaction without PVD are not different from oxygen levels in animals with neither vitreous liquefaction nor PVD [30]. Our understanding that the posterior vitreous consumes oxygen at a higher rate adds to Quiram’s findings. We believe that the vitreous cortex does not slow down the process of diffusion by having a slower diffusion constant, but rather, consumes oxygen at a greater rate than the rest of the vitreous.

### Conclusion

In this study, we investigated the spatial variation of vitreous oxygen consumption in enucleated porcine eyes. We fabricated a custom oxygen source, implanted it in the mid vitreous and the posterior vitreous and found that the oxygen concentration profiles were statistically different between these two locations; suggesting a spatial variation in vitreous oxygen consumption. In conjunction with that finding, we observed statistically different concentrations of ascorbate across the two locations and quantified the reaction rate between ascorbate and oxygen in vitreous.

## Supporting Information

S1 Appendix Point source diffusion equation. (DOCX)

## References

[1] Foulds WS (1987) Is your vitreous really necessary? The role of the vitreous in the eye with particular reference to retinal attachment, detachment and the mode of action of vitreous substitutes. Eye (Lond) 1 (Pt 6): 641–664. Available: http://www.ncbi.nlm.nih.gov/pubmed/3331605. Accessed 3 November 2014. 3331605 10.1038/eye.1987.107

[2] Sebag J (1987) Ageing of the vitreous. Eye (Lond) 1 (Pt 2): 254–262. Available: http://www.ncbi.nlm.nih.gov/pubmed/3308528. Accessed 10 August 2014. 3308528 10.1038/eye.1987.45

[3] Siegfried CJ, Shui Y-B, Holekamp NM, Bai F, Beebe DC (2010) Oxygen distribution in the human eye: relevance to the etiology of open-angle glaucoma after vitrectomy. Invest Ophthalmol Vis Sci 51: 5731–5738. Available: http://www.pubmedcentral.nih.gov/articlerender.fcgi?artid=3061509&tool=pmcentrez&rendertype=abstract. Accessed 25 June 2012. 10.1167/iovs.10-5666 20720218 PMC3061509

[4] Ernest JT, Archer DB (1979) Vitreous body oxygen tension following experimental branch retinal vein obstruction. Invest Ophthalmol Vis Sci 18: 1025–1029. Available: http://www.ncbi.nlm.nih.gov/pubmed/90026. 90026

[5] Sakaue H, Negi A, Honda Y (1989) Comparative study of vitreous oxygen tension in human and rabbit eyes. Invest Ophthalmol Vis Sci 30: 1933–1937. Available: http://www.iovs.org/content/30/9/1933.short. Accessed 23 October 2014. 2777513

[6] Yu DY, Cringle SJ (2001) Oxygen distribution and consumption within the retina in vascularised and avascular retinas and in animal models of retinal disease. Prog Retin Eye Res 20: 175–208. Available: http://www.ncbi.nlm.nih.gov/pubmed/11173251. 11173251 10.1016/s1350-9462(00)00027-6

[7] Wangsa-Wirawan ND, Linsenmeier RA (2003) Retinal oxygen: fundamental and clinical aspects. Arch Ophthalmol 121: 547–557. Available: http://www.ncbi.nlm.nih.gov/pubmed/12695252. Accessed 23 October 2014. 12695252 10.1001/archopht.121.4.547

[8] Stefánsson E (2009) Physiology of vitreous surgery. Graefes Arch Clin Exp Ophthalmol 247: 147–163. Available: http://www.ncbi.nlm.nih.gov/pubmed/19034481. Accessed 29 May 2012. 10.1007/s00417-008-0980-7 19034481

[9] Stefánsson E, Loftsson T (2006) The Stokes-Einstein equation and the physiological effects of vitreous surgery. Acta Ophthalmol Scand 84: 718–719. Available: http://www.ncbi.nlm.nih.gov/pubmed/17083526. Accessed 29 May 2013. 17083526 10.1111/j.1600-0420.2006.00778.x

[10] Shui YY-B, Holekamp NMNNM, Kramer BC, Crowley JR, Wilkins MA, Chu F, et al (2009) The gel state of the vitreous and ascorbate-dependent oxygen consumption: relationship to the etiology of nuclear cataracts. Arch Ophthalmol 127: 475–482. Available: http://archpsyc.jamanetwork.com/article.aspx?articleid=422840. Accessed 23 October 2014. 10.1001/archophthalmol.2008.621 19365028 PMC2683478

[11] Beebe DC, Shui Y-B, Siegfried CJ, Holekamp NM, Bai F (2014) Preserve the (intraocular) environment: the importance of maintaining normal oxygen gradients in the eye. Jpn J Ophthalmol 58: 225–231. Available: http://www.ncbi.nlm.nih.gov/pubmed/24687817. Accessed 12 August 2014. 10.1007/s10384-014-0318-4 24687817

[12] Skeie JM, Mahajan VB (2011) Dissection of human vitreous body elements for proteomic analysis. J Vis Exp: e2455 Available: http://www.jove.com/video/2455. Accessed 3 October 2014. 10.3791/2455 PMC3182653 21304469

[13] Locci E, Scano P, Rosa MF, Nioi M, Noto A, Atzori L, et al (2014) A metabolomic approach to animal vitreous humor topographical composition: a pilot study. PLoS One 9: e97773 Available: http://www.pubmedcentral.nih.gov/articlerender.fcgi?artid=4028277&tool=pmcentrez&rendertype=abstract. Accessed 23 October 2014. 10.1371/journal.pone.0097773 24845217 PMC4028277

[14] Abdallah W, Ameri H, Barron E, Chader GJ, Greenbaum E, Hinton D, et al (2011) Vitreal oxygenation in retinal ischemia reperfusion. Invest Ophthalmol Vis Sci 52: 1035–1042. Available: http://www.pubmedcentral.nih.gov/articlerender.fcgi?artid=3053094&tool=pmcentrez&rendertype=abstract. Accessed 3 October 2014. 10.1167/iovs.09-4516 21051734 PMC3053094

[15] Kang D, Murali K, Scianmarello N, Park J, Chang JH-C, Liu Y et al. (2015) MEMS oxygen transporter to treat retinal ischemia. 2015 28th IEEE International Conference on Micro Electro Mechanical Systems (MEMS). IEEE. pp. 154–157. Available: http://ieeexplore.ieee.org/lpdocs/epic03/wrapper.htm?arnumber=7050909.

[16] EISON-PERCHONOK MH, DOWNES TW (1982) Kinetics of Ascorbic Acid Autoxidation as a Function of Dissolved Oxygen Concentration and Temperature. J Food Sci 47: 765–767. Available: http://onlinelibrary.wiley.com/doi/10.1111/j.1365-2621.1982.tb12710.x/abstract. Accessed 27 November 2014.

[17] Filas B a, Shui Y-B, Beebe DC (2013) Computational model for oxygen transport and consumption in human vitreous. Invest Ophthalmol Vis Sci 54: 6549–6559. Available: http://www.pubmedcentral.nih.gov/articlerender.fcgi?artid=3797591&tool=pmcentrez&rendertype=abstract. Accessed 15 April 2014. 10.1167/iovs.13-12609 24008409 PMC3797591

[18] Shui Y-B, Fu J-J, Garcia C, Dattilo LK, Rajagopal R, McMillan S, et al (2006) Oxygen distribution in the rabbit eye and oxygen consumption by the lens. Invest Ophthalmol Vis Sci 47: 1571–1580. Available: http://www.ncbi.nlm.nih.gov/pubmed/16565394. Accessed 25 June 2012. 16565394 10.1167/iovs.05-1475

[19] Padh H (1991) Vitamin C: newer insights into its biochemical functions. Nutr Rev. Available: http://onlinelibrary.wiley.com/doi/10.1111/j.1753-4887.1991.tb07407.x/abstract. Accessed 24 October 2014. 10.1111/j.1753-4887.1991.tb07407.x 2057141

[20] Rose RC, Richer SP, Bode a. M (1998) Ocular Oxidants and Antioxidant Protection. Exp Biol Med 217: 397–407. Available: http://ebm.sagepub.com/lookup/doi/10.3181/00379727-217-44250. Accessed 12 August 2014. 10.3181/00379727-217-44250 9521086

[21] Rose RC, Bode AM (1991) Ocular ascorbate transport and metabolism. Comp Biochem Physiol Part A Physiol 100: 273–285. Available: http://www.sciencedirect.com/science/article/pii/030096299190470W. Accessed 27 November 2014. 10.1016/0300-9629(91)90470-w 1685949

[22] Eaton JW (1991) Is the lens canned? Free Radic Biol Med 11: 207–213. Available: http://linkinghub.elsevier.com/retrieve/pii/089158499190173Z. Accessed 24 October 2014. 1937139 10.1016/0891-5849(91)90173-z

[23] Silverblatt E, Robinson A, King C (1943) The kinetics of the reaction between ascorbic acid and oxygen in the presence of copper ion. J Am … 455 Available: http://pubs.acs.org/doi/abs/10.1021/ja01242a002. Accessed 23 October 2014.

[24] Cabelli DE, Bielski BHJ (1983) Kinetics and mechanism for the oxidation of ascorbic acid/ascorbate by HO2/O2- (hydroperoxyl/superoxide) radicals. A pulse radiolysis and stopped-flow photolysis study. J Phys Chem 87: 1809–1812. Available: http://pubs.acs.org/doi/abs/10.1021/j100233a031. Accessed 27 November 2014.

[25] Johnson SW (1936) Cataract and ascorbic acid in the guinea-pig eye. Biochem J 30: 1430–1437. Available: http://www.pubmedcentral.nih.gov/articlerender.fcgi?artid=1263202&tool=pmcentrez&rendertype=abstract. 16746175 10.1042/bj0301430 PMC1263202

[26] DiMattio J (1989) A comparative study of ascorbic acid entry into aqueous and vitreous humors of the rat and guinea pig. Invest Ophthalmol Vis Sci 30: 2320–2331. Available: http://www.iovs.org/content/30/11/2320.short. Accessed 23 October 2014. 2807790

[27] Behndig A, Svensson B, Marklund SL, Karlsson K (1998) Superoxide dismutase isoenzymes in the human eye. Invest Ophthalmol Vis Sci 39: 471–475. Available: http://www.iovs.org/content/39/3/471.short. Accessed 26 November 2014. 9501855

[28] Tsukaguchi H, Tokui T, Mackenzie B, Berger U V, Chen XZ, Wang Y, et al (1999) A family of mammalian Na+-dependent L-ascorbic acid transporters. Nature 399: 70–75. Available: http://www.ncbi.nlm.nih.gov/pubmed/10331392. Accessed 12 October 2014. 10331392 10.1038/19986

[29] Salceda R, Contreras-Cubas C (2007) Ascorbate uptake in normal and diabetic rat retina and retinal pigment epithelium. Comp Biochem Physiol Part C Toxicol Pharmacol 146: 175–179. 10.1016/j.cbpc.2007.02.015 17395543

[30] Quiram PA, Leverenz VR, Baker RM, Dang L, Giblin FJ, Trese MT, et al (2007) Microplasmin-induced posterior vitreous detachment affects vitreous oxygen levels. Retina 27: 1090–1096. Available: http://www.ncbi.nlm.nih.gov/pmc/articles/PMC2702988/. Accessed 3 October 2014. 18040251 10.1097/IAE.0b013e3180654229 PMC2702988

