# Supplementary material for: Spatial Variations in Vitreous Oxygen Consumption
Source: PLoS One. 2016 Mar 1;11(3):e0149961. doi: 10.1371/journal.pone.0149961 (PMC4773158; doi:10.1371/journal.pone.0149961)
Supplement: S1 Appendix — (DOCX) [file pone.0149961.s001.docx]

**S1 Appendix**

Point source diffusion equation. In this equation, we are modeling the concentration of oxygen as it spreads in a 3-dimensional sphere away from a point source.

C refers to Concentration in M (Molar)

r refers to Radius in mm

D refers to Diffusion Coefficient in mm^2^ s^-1^

$$\frac{dC}{dr}=\frac{D}{r^{2}}\frac{d}{dr}\left( r^{2}\frac{dC}{dr} \right)+R (1)$$

$$\frac{D}{r^{2}}\frac{d}{dr}\left( r^{2}\frac{dC}{dr} \right)=-R (2)$$

Reaction equation between ascorbate and oxygen.

AH^—^refers to Ascorbate

A refers to Dehydroascorbate

R refers to Reaction Rate

k refers to the Reaction Rate Constant

$${AH}^{-}+O_{2}+H^{+}\to A+H_{2}O_{2} (3)$$

$$R=\frac{dO_{2}}{dt}=-k[{AH}^{-}][O_{2}] (4)$$

$$\frac{d{[AH}^{-}]}{dt}=0 (5)$$

$$\ln[O_{2}]=\ln{{[O}_{2}]}_{0}-\acute{k}t (6)$$

$$where \acute{k}=k({AH}^{-})$$

Plug (4) into (2)

$$\frac{D}{r^{2}}\frac{d}{dr}\left( r^{2}\frac{dC}{dr} \right)=k({AH}^{-}) C (7)$$

$$\frac{D(2\frac{dC}{dr}+r\frac{d^{2}C}{{dr}^{2}})}{r}= k({AH}^{-}) C (8)$$

Let

$$C=\frac{v(r)}{r} (9)$$

Thus,

$$\frac{dC}{dr}=\frac{\frac{dv}{dr}}{r}-\frac{v}{r^{2}} (10)$$

$$\frac{d^{2}C}{{dr}^{2}}=\frac{2v}{r^{3}}+\frac{\frac{d^{2}v}{{dr}^{2}}}{r}-\frac{2\frac{dv}{dr}}{r^{2}} (11)$$

Plug into (9), (10), and (11) into (8)

$$D\frac{d^{2}v}{{dr}^{2}}=k({AH}^{-}) v (12)$$

Let

$$v=e^{\lambda r} (13)$$

Substitute (13) into (12)

$$\left( -\left( k\left( {AH}^{-} \right) \right)+D\lambda^{2} \right)e^{\lambda r}=0 (14)$$

$$\lambda=\pm\frac{\sqrt{k\left( {AH}^{-} \right)}}{\sqrt{D}} (15)$$

General solution is the sum of the above solutions

$$v=v_{1}+v_{2} (16)$$

Where

$$v_{1}=c_{1}e^{-\frac{\sqrt{k\left( {AH}^{-} \right)}}{\sqrt{D}}r} (17)$$

$$v_{2}=c_{2}e^{+\frac{\sqrt{k\left( {AH}^{-} \right)}}{\sqrt{D}}r} (18)$$

$$v=c_{1}e^{-\frac{\sqrt{k\left( {AH}^{-} \right)}}{\sqrt{D}}r}+c_{2}e^{+\frac{\sqrt{k\left( {AH}^{-} \right)}}{\sqrt{D}}r} (19)$$

Substitute back for C

$$rC=c_{1}e^{-\frac{\sqrt{k\left( {AH}^{-} \right)}}{\sqrt{D}}r}+c_{2}e^{+\frac{\sqrt{k\left( {AH}^{-} \right)}}{\sqrt{D}}r} (20)$$

$$C\left( r \right)=\frac{c_{1}e^{-r\sqrt{\frac{{AH}^{-}k}{D}}}}{r}+\frac{c_{2}e^{r\sqrt{\frac{{AH}^{-}k}{D}}}}{r\sqrt{\frac{{AH}^{-}k}{D}}} (21)$$

This equation describes the concentration profile of oxygen as we move away from the point source.
